# Establishment of a medium-scale mosquito facility: optimization of the larval mass-rearing unit for *Aedes albopictus* (Diptera: Culicidae)

**DOI:** 10.1186/s13071-017-2511-z

**Published:** 2017-11-13

**Authors:** Dongjing Zhang, Meichun Zhang, Yu Wu, Jeremie R. L. Gilles, Hanano Yamada, Zhongdao Wu, Zhiyong Xi, Xiaoying Zheng

**Affiliations:** 1 Department of Parasitology, Zhongshan School of Medicine, Sun Yat-sen University, Guangzhou, Guangdong 510080 China; 2 Key Laboratory for Tropical Disease Control, Ministry of Education, Sun Yat-sen University, Guangzhou, Guangdong 510080 China; 3 Guangdong Provincial Engineering Technology Research Center for Diseases-vectors Control, Sun Yat-sen University, Guangzhou, Guangdong 510080 China; 4 Sun Yat-sen University - Michigan State University Joint Center of Vector Control for Tropical Diseases, Zhongshan School of Medicine, Guangzhou, Guangdong 510080 China; 5 Insect Pest Control Laboratory, Joint FAO/IAEA Division of Nuclear Techniques in Food and Agriculture, A1130 Vienna, Austria; 6 Department of Microbiology and Molecular Genetics, Michigan State University, East Lansing, MI 48824 USA

**Keywords:** Mosquito facility, Larval rearing units, Mass-rearing, *Aedes albopictus*

## Abstract

**Background:**

Standardized larval rearing units for mosquito production are essential for the establishment of a mass-rearing facility. Two larval rearing units, developed respectively by the Guangzhou Wolbaki Biotech Co. Ltd. (Wolbaki) and Insect Pest Control Laboratory, Joint FAO/IAEA Division of Nuclear Techniques in Food and Agriculture (FAO/IAEA-IPCL), are tested to assess their potential uses to mass-rear the larval stages of *Aedes albopictus* in support of the establishment of a medium-scale mosquito facility for the application of mosquito genetic control strategies.

**Methods:**

The triple *Wolbachia*-infected *Ae. albopictus* strain (HC strain) was used in this study. The effects of larval densities of two larval rearing trays (corresponding to 2.4, 3.0 and 3.6 larvae/cm^2^) and tray size/position (top, middle and bottom layers) on the pupae production and larval survival were assessed when trays were stacked within the larval rearing units. The male pupae production, female pupae contamination after sex separation, and male mating competitiveness were also studied by using both larval rearing units in their entirety.

**Results:**

The optimal larval rearing density for Wolbaki-tray (Wol-tray) was 6,600 larvae (equal to 3.0 larvae/cm^2^) and 18,000 larvae (3.6 larvae/cm^2^) for the FAO/IAEA-IPCL tray (IAEA-tray). No significant difference in pupae production was observed when trays were stacked within top, middle or bottom layers for both units. At thirty-four hours after the first pupation, the average male pupae production was (0.89 × 10^5^) for the Wol-unit and (3.16 × 10^5^) for the IAEA-unit. No significant difference was observed in female pupae contamination between these two units. The HC males showed equal male mating competitiveness to wild type males for mating with wild type females in large cages, regardless of whether they were reared in the Wol-unit or IAEA-unit.

**Conclusions:**

The current study has indicated that both the Wol-unit and IAEA-unit are suitable for larvae mass-rearing for *Ae. albopictus.* However, the IAEA-unit, with higher male production and less space required compared to the Wol-unit, is recommended to be used in support of the establishment of a medium-sized mosquito facility.

**Electronic supplementary material:**

The online version of this article (10.1186/s13071-017-2511-z) contains supplementary material, which is available to authorized users.

## Background

*Aedes albopictus* transmits many pathogens (mainly belonging to the family *Flaviviridae*), including dengue virus and chikungunya virus [1–3], as well as Zika virus [4], which has been linked to the rise in microcephaly incidences in Brazil in 2016 [5]. Traditional mosquito control methods, such as insecticide applications and source reduction, are insufficient to sustainably control this invasive mosquito species [6–8]. Thus, novel strategies and techniques are being considered to fight these mosquitoes, such as the sterile insect technique (SIT), the incompatible insect technique (IIT), or a combination of both techniques [9–13]. Both of these techniques are based on the inundated release of large numbers of high quality sterile male mosquitoes (to compete with their wild male counterparts) to mate with wild females in a target area, thus inducing female sterility which results in a reduction in the population. The aim is to reduce or prevent the transmission of mosquito borne diseases [10, 14]. Both the SIT and IIT, as a component of area-wide integrated pest management (AW-IPM) programs, depend on several important steps including mass-rearing, sex separation, sterilization, transportation, release and monitoring [15].

In a mosquito SIT or IIT program, release of males only improves the efficiency of population suppression and reduces potential disease transmission by accidentally released females [16], although *Wolbachia*-infected females already have a reduced ability to transmit pathogens [17, 18]. In the case of IIT, which depends on *Wolbachia*-induced cytoplasmic incompatibility (CI), the inadvertent release of *Wolbachia*-infected females might lead to population replacement, resulting in failure in population suppression if males carrying the same strain of *Wolbachia* are used for further release. One of the strategies to eliminate the risk of population replacement is to use a low dosage of irradiation to completely sterilize the females [19], while not negatively affecting male mating competitiveness and the strength of CI [20]. As stated by the WHO, this combined SIT/IIT technology has potential for long-term control of *Aedes aegypti* and *Ae. albopictus* mosquitoes [21]. Previous studies have indicated that the combined SIT/IIT approach using a triple *Wolbachia*-infected strain (HC) is considered to be an effective and safe strategy to control *Ae. albopictus* [19, 20, 22].

*Aedes albopictus* is considered to be suitable for SIT/IIT application because of its intermittent distribution, short flight range and ease of monitoring, even at low population densities [23]. In addition, during sterile male production, females can be easily removed from the release material through the Fay-Morlan glass separator with a 99% sex separation efficiency due to the species’ natural protandry and physical size difference between male and female pupae [24, 25]. The current genetic control strategies, including *Wolbachia*-based, irradiation-based and transgenic mosquito-based approaches, require standardized rearing methods for both larval and adult life stages in order to produce enough sterile males of high quality. The larval rearing is affected by several factors, such as the larval rearing density [25, 26], water temperature [25], water depth [24, 25], food quality and quantity [24, 26] as well as the structure of the rearing tray [25]. Many studies have been carried out on the optimization of *Ae. albopictus* mass-rearing protocols by improving rearing methods (such as finding suitable larval diets for mass-rearing) [27] and developing new rearing units for both larvae [24–26, 28] and adults [29].

To mass-rear mosquito larval stages, the FAO/IAEA Insect Pest Control Laboratory (FAO/IAEA-IPCL) has developed a larval rearing unit (IAEA-unit) which consists of a mechanized stainless steel rack that can hold 50 rearing trays with the estimated capacity to produce 100,000 male pupae by using a sieve sorting method for separating male and female pupae [25, 28]. The Wolbaki has also developed a larval rearing unit (Wol-unit) which consists of a mechanized stainless steel rack that can hold 40 rearing trays. Our study aims to evaluate the effects of rearing density and tray position (height, associated with a temperature gradient in the room) of both larval rearing units on pupae production and larval survival. In addition, the pupae production, female pupae contamination and male mating competitiveness were also assessed by using either the IAEA- or Wol-units. By comparison of these two larval rearing units, we aim to find the suitable larval rearing unit for varying scales of mosquito mass-rearing facilities. Our study also provides valuable information on the optimization of the larval rearing methods in a mosquito facility setting.

## Methods

### Mosquito strain and rearing conditions

Two different *Wolbachia*-infected *Ae. albopictus* strains were used in this experiment: the triple *Wolbachia*-infected HC strain (*w*AlbA, *w*AlbB and *w*Pip) and the wild type GUA strain (*w*AlbA and *w*AlbB) [22]. The HC strain, also maintained at the FAO/IAEA-IPCL [22], has been mass-reared in the Wolbaki facility for two years while the GUA strain has been maintained under laboratory conditions for nearly three years. Mosquitoes were maintained and experiments conducted in a climate-controlled room at 28 ± 1 °C, 80 ± 10% RH, and a photoperiod of 12:12 h (L:D). The gradient temperature in the mosquito rearing room at vertical positions (from floor to height at 2 m) was less than 1 °C, which was measured by the Testo logger (175 H1, Schwarzwald, Germany).

### Effects of larval rearing density on pupae production and larval survival

**Fig. 1:**
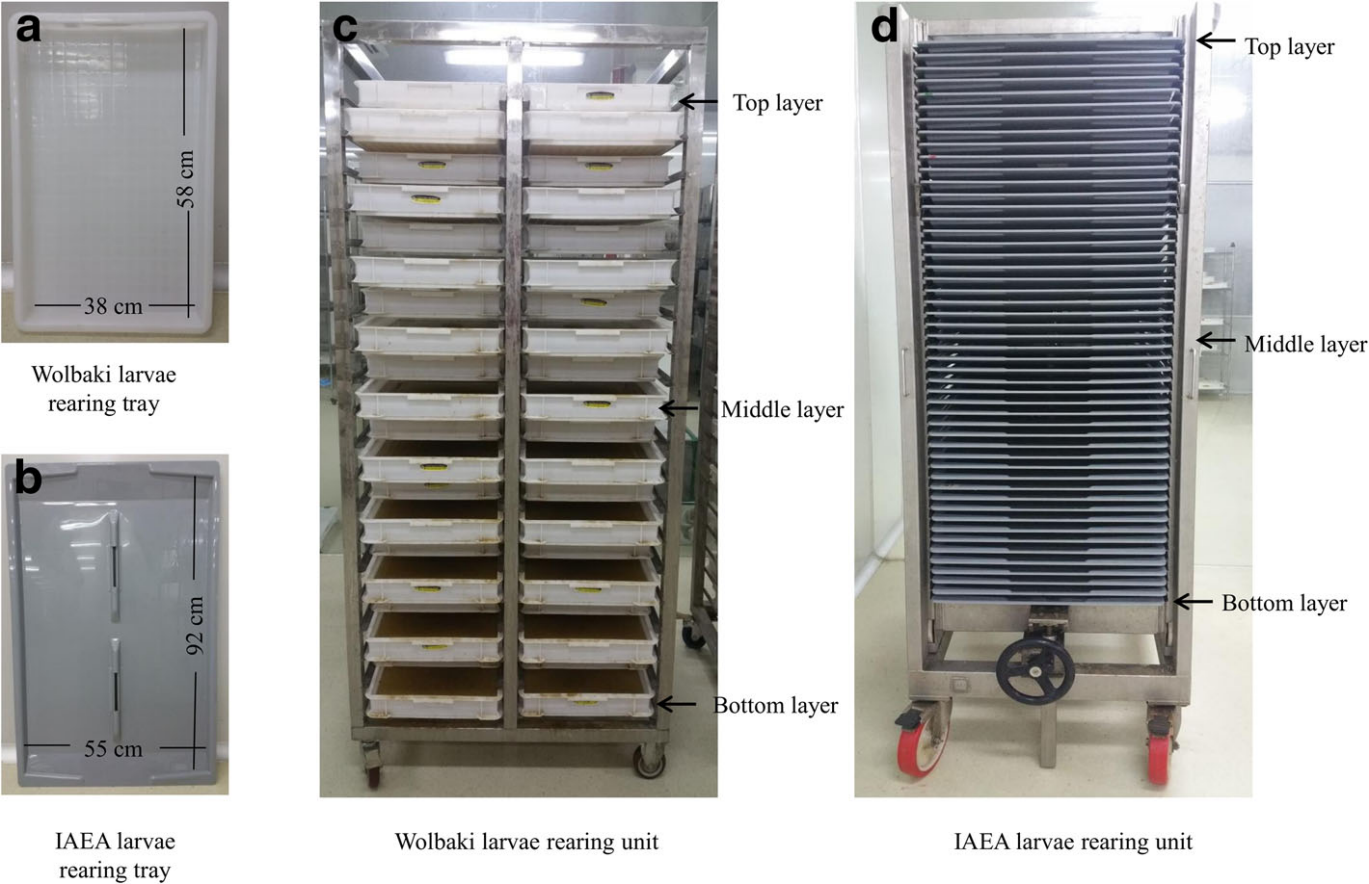
Larval mass-rearing tray and unit. **a** Wolbaki larval rearing tray (inner surface ≈ 2200 cm^2^). **b** IAEA larval rearing tray (inner surface ≈ 5000 cm^2^). **c** Wolbaki larval rearing unit with 40 trays (1.85 m height). **d** IAEA larval rearing unit with 50 trays (2.10 m height)

Two different plastic larval rearing trays were used to test the effects of rearing density on pupae production and larvae survival of HC strain, including the first model of the Wolbaki larval rearing tray (Wol-tray: L × W × H = 58 × 38 × 4 cm, Guangzhou, China) (Fig. 1a) and the IAEA larval rearing tray (IAEA-tray: L × W × H = 92 × 55 × 2 cm, Voesendorf, Austria) (Fig. 1b). The available inner surface of these two trays was approximately 2200 cm^2^ for the Wol-tray and 5000 cm^2^ for the IAEA-tray. Three different larval rearing densities were assessed for both trays: 2.4, 3.0 and 3.6 larvae/cm^2^, corresponding to 5300, 6600 and 7900 larvae (L_1_) for the Wol-tray and 12,000, 15,000 and 18,000 larvae for the IAEA-tray. The Wol-trays and the IAEA-trays held approximately 2.6 l and 6.0 l water, respectively, arriving at a water depth of 1.2 cm for both trays. The larvae were fed daily on larvae diet (Bovine liver powder 50%, Shrimp powder 30%, and Yeast powder 20%) (Wolbaki Biotech Co, Ltd., Guangzhou, China) according to Zhang et al. [22]. Larval diet (6.0%) was provided as: 0.21 mg/larva, 0.21 mg/larva, 0.84 mg/larva, 1.26 mg/larva, 1.68 mg/larva and 0.84 mg/larva from day 1 to 6, respectively. In order to reduce the potential impact of temperature variations on pupae production and larval survival, all tests were performed in trays held at the same height (around 1.6 m from the floor). Three replicates were performed for each rearing density.

Pupae were separated from larvae by using a modified Fay-Morlan separator [30] and counted individually at 34 h (± 2 h) after the first pupation. The remaining larvae returned to the original rearing tray and pupae and larvae were collected and counted again 24 h later (collection time = 58 h).

### Effects of the height of larval rearing trays in the unit on pupae production and larval survival

The height of the Wolbaki larval rearing unit (Wol-unit) and the FAO/IAEA-IPCL larval rearing unit (IAEA-unit) was 1.85 m and 2.10 m, respectively. The Wol-unit can hold 40 trays, while the IAEA-unit can hold 50 trays. Since we observed a temperature gradient associated with the increasing height of the larval rearing trays, and the air temperature influences the water temperature and thus has an impact on the larval development, we divided both units into three sections: top, middle and bottom layers (Fig. 1c, d). Based on the study performed above, we selected the most optimal larval rearing density which was 6600 L_1_ for Wol-tray and 18,000 L_1_ for IAEA-tray, which gave the maximum pupae without affecting the percentage of male pupae. The amount of diet per larva and per day at different developmental stages was identical to the above study. Pupae were separated from larvae at 34 h (± 2 h) after the beginning of the pupation period (first pupa). Pupae and larvae were collected and recorded individually. Three replicates were performed for each section. The water temperature of each tray was recorded every day until pupae separation.

### Male pupae production, female pupae contamination and male mating competitiveness following the use of the two different larval rearing units

Under the optimal larval rearing density described above, the male pupae production, female pupae contamination and male mating competitiveness were assessed after using either the Wolbaki or IAEA larval rearing units. This meant that approximately a total of 2.64 × 10^5^ L_1_ were reared for the entire Wol-unit and 9.0 × 10^5^ L_1_ for the IAEA-unit. The rearing methods and separation time were the same as described above. The male pupae production was estimated volumetrically. Three hundred to 1000 of the obtained male pupae were randomly selected and sexed under the stereomicroscope to assess the extent of female pupae contamination. Male pupae production efficiency of a rearing unit was calculated as the number of male pupae collected divided by the estimated average of male pupae production acquired from the top, middle and bottom sections of the larval rearing unit. Five replicates were performed for both larval rearing units.

The male mating competitiveness of HC males, which were reared in either the Wol-unit or the IAEA-unit, was assessed at a 1:1 ratio of sterile to fertile GUA males, competing for GUA females in large brown cylindrical cages (2.9 × 2.9 × 2.0 m, 13.2 m^3^). Male pupae from either the Wol-unit or the IAEA-unit were randomly selected for this mating experiment. One hundred GUA males and 100 virgin GUA females were used in all treatment cages. Control cages were also included: the sterile control with 50 virgin GUA females paired with 50 sterile males (Wol-unit HC or IAEA-unit HC males), and the fertile controls with 50 virgin GUA females paired with 50 fertile GUA males. The competitive mating experiments were performed according to a previous study by Zhang et al. [20]. Three replicates were performed for both controls and treatment cages.

### Data collected and statistical analysis

Several equations calculated in this study are shown below:

(i) Larval survival of each tray was calculated as: (Total number of pupae collected + Residual number of larvae after sex separation)/(Estimated number of larvae per tray);
(ii) Estimated number of male pupae per tray was calculated as the average male pupae at different heights of the units;
(iii) Estimated male pupae production per unit was calculated as: (Number of trays per unit × Estimated number of male pupae per tray);
(iv) Male pupae production efficiency was calculated as: (Male pupae production per unit / Estimated male pupae production);
(v) Male mating competitiveness index (C) was calculated as: C = [(Hn – Ho) / (Ho – Hs)] × (N / S), where Hn is the hatch rate of the fertile controls, Ho is the observed egg hatch rate from each competition cage and Hs is the hatch rate of the sterile controls (with HC males reared either in the Wol-unit or the IAEA-unit). N and S are the numbers of fertile and sterile males, respectively [31].

Analyses were conducted using Graph Pad Prism 6.0 software. The percentage of male pupae, female pupae contamination rate and male pupae production efficiency were arcsin-transformed. Normality of the data was assessed by the D’Agostino-Pearson omnibus normality test. One-way analysis of variance (ANOVA) and Tukey *post-hoc* test were used to compare the differences in the number of pupae produced at 34 and 58 h from the onset of pupation, the number of male pupae, the percentage of male pupae and the larval survival according to larval rearing densities as well as the height of larval rearing unit. Mann-Whitney U-test was used to compare the female pupae contamination rate, male pupae production efficiency and male mating competitiveness index between the Wol-unit and the IAEA-unit.

## Results

### Effects of larval rearing densities on pupae production and larval survival

A significant difference was observed in pupae production from different larval rearing densities for both the Wol-tray and the IAEA-tray at 34 h (Wol-tray: *F*_(2, 6)_ = 192.4, *P* < 0.0001; IAEA-tray: *F*_(2, 6)_ = 176.2, *P* < 0.0001) and 58 h (Wol-tray: *F*_(2, 6)_ = 211.9, *P* < 0.0001; IAEA-tray: *F*_(2, 6)_ = 26.2, *P* = 0.0011) after pupation, respectively (Table 1). However, no significant difference was observed for pupae production between densities of 6600 and 7900 L_1_ per Wol-tray at 34 h (Tukey *post-hoc* test, *P* > 0.05) or between densities of 15,000 and 18,000 L_1_ per IAEA-tray at 58 h (Tukey *post-hoc* test, *P* > 0.05) (Table 1). A significant difference was observed in male pupae production from the three tested larval rearing densities for both the Wol-tray at 34 h and 58 h (34 h: *F*_(2, 6)_ = 41.7, *P* = 0.0003; 58 h: *F*_(2, 6)_ = 26.7, *P* = 0.0010) and the IAEA-tray at 34 h (*F*_(2, 6)_ = 24.9, *P* = 0.0012); however, this difference was not observed for the IAEA-tray at 58 h (*F*_(2, 6)_ = 3.5, *P* = 0.1000) (Table 1). At 34 h after the first pupation, the male pupae production was not statistically different between densities of 6600 and 7900 L_1_ for the Wol-tray (Tukey *post-hoc* test, *P* > 0.05), while a higher number of male pupae was achieved at a density of 18,000 L_1_ in the IAEA-tray compared to the other two densities (Tukey *post-hoc* test, *P* < 0.05) (Table 1). For the IAEA-tray, the recommended larval rearing density is therefore 18,000 L_1_ per tray, which produced a higher number of male pupae than the other two rearing densities at 34 h of pupation (Table 1). For the Wol-tray, the favorable density was 6600 L_1_ per tray, which did not produce a statistically different number of male pupae to the density of 7900 L_1_ per tray (Table 1).

**Table 1:** Effects of *Aedes albopictus* larval rearing densities on the pupae production and larval survival at different pupation times when using the Wol-tray or the IAEA-tray

Tray	Number of larvae per tray	34 h (Mean ± SE)			58 h (Mean ± SE)			Larval survival (%)^a^ (Mean ± SE)
		PP	MPP	M%	PP	MPP	M%	
Wol-tray	5300	3092 ± 27 a	2396 ± 46 a	70.0 ± 1.9 a	1252 ± 60 a	358 ± 53 a	25.6 ± 2.8 ab	95.2 ± 2.4 a
	6600	3986 ± 62 b	3003 ± 29 b	72.8 ± 0.5 a	1798 ± 15 b	345 ± 22 a	18.5 ± 0.9 a	99.0 ± 0.4 a
	7900	4152 ± 22 b	3188 ± 98 b	74.2 ± 1.1 a	2312 ± 10 c	662 ± 19 b	27.7 ± 1.1 b	96.1 ± 1.0 a
IAEA-tray	12,000	6752 ± 192 A	5238 ± 63 A	76.3 ± 0.4 A	3306 ± 196 A	712 ± 60 A	21.1 ± 0.7 A	97.9 ± 1.0 A
	15,000	8240 ± 31 B	5961 ± 124 A	71.1 ± 0.6 B	4540 ± 77 B	937 ± 52 A	20.2 ± 0.4 A	98.4 ± 0.8 A
	18,000	10,281 ± 125 C	7349 ± 346 B	70.2 ± 1.6 B	4335 ± 78 B	883 ± 76 A	20.0 ± 1.1 A	97.7 ± 1.0 A

*Abbreviations*: *PP* pupae production, *MPP* male pupae production, *M%* percentage of male pupae in PP

^a^Larval survival was calculated as: (Total number of pupae collected + Residual number of larvae after sex separation) / (Estimated number of larvae per tray)

*Note*: Within each column, values followed by different lowercase or capital letters were statistically different using ANOVA analysis and Tukey *post-hoc* test (*P* < 0.05)

There was no significant difference for the percentage of male pupae out of the total pupae production for the Wol-tray at 34 h (*F*_(2, 6)_ = 2.7, *P* = 0.1493) and for the IAEA-tray at 58 h (*F*_(2, 6)_ = 0.5, *P* = 0.6050), respectively (Table 1). The percentage of male pupae in a larval rearing density of 12,000 L_1_ had a significantly higher value than that for densities of 15,000 and 18,000 L_1_ at 34 h (*F*_(2, 6)_ = 10.8, *P* = 0.0103) (Table 1). No significant difference was observed for larval survival for both the Wol-tray and the IAEA-tray, (Wol-tray: *F*_(2, 6)_ = 2.4, *P* = 0.1700; IAEA-tray: *F*_(2, 6)_ = 0.2, *P* = 0.7946) (Table 1).

### Effects of height of larval rearing trays in units on pupae production and larval survival

As shown in Table 2, the IAEA-unit had a higher water temperature (around 1–1.5 °C) than the Wol-unit under the same rearing conditions. The top layer of both units had a higher water temperature than both the middle and bottom layers (Table 2). The larval rearing density was 6600 larvae per tray for the Wol-unit and 18,000 larvae per tray for the IAEA-unit. No significant difference was observed on pupae production among the tested top, middle and bottom layers in both Wol-uint and IAEA-unit (Table 2) (Wol-unit: *F*_(2, 6)_ = 2.9, *P* = 0.1335; IAEA-unit: *F*_(2, 6)_ = 0.9, *P* = 0.4557). No significant difference was observed on the percentage of male pupae in the total pupae production (Wol-tray: *F*_(2, 6)_ = 0.4, *P* = 0.7000; IAEA-tray: *F*_(2, 6)_ = 0.4, *P* = 0.7006) and larval survival (Wol-tray: *F*_(2, 6)_ = 0.2, *P* = 0.8241; IAEA-tray: *F*_(2, 6)_ = 0.3, *P* = 0.7433) when using both units under the optimal rearing density (Table 2).

**Table 2:** Effects of the height of the larval rearing trays in units on pupal production and larval survival

Unit	Layers of unit	Water temp (°C)	34 h (Mean ± SE)		
			PP	M%	Larval survival (%)^a^
Wol-unit	Top	26.7 ± 0.2	3399 ± 101 a	76.9 ± 1.6 a	99.3 ± 1.9 a
	Middle	26.4 ± 0.2	3247 ± 137 a	77.2 ± 2.0 a	98.5 ± 1.3 a
	Bottom	26.2 ± 0.1	2873 ± 219 a	79.2 ± 2.4 a	98.3 ± 0.8 a
IAEA-unit	Top	28.4 ± 0.2	10,390 ± 704 A	73.5 ± 0.7 A	98.3 ± 0.4 A
	Middle	27.4 ± 0.3	9407 ± 552 A	75.0 ± 1.3 A	96.9 ± 3.5 A
	Bottom	27.7 ± 0.3	9100 ± 847 A	74.5 ± 1.7 A	96.6 ± 0.5 A

^a^Larval survival was calculated as: (Total number of pupae collected + Residual number of larvae after sex separation) / (Estimated number of larvae per tray)

*Note*: Within each column, values followed by different lowercase or capital letters were statistically different using ANOVA analysis and Tukey *post-hoc* test (*P* < 0.05)

*Abbreviations*: *PP* pupae production, *M%* percentage of male pupae in PP

### Male pupae production, female pupae contamination and male mating competitiveness following the use of the entire larval rearing units

At 34 h after the first pupation, the average male pupae production after using the entire larval rearing unit was 0.89 × 10^5^ for Wol-unit and 3.16 × 10^5^ for IAEA-unit under their respective optional larval rearing density (Table 3). No significant difference was observed in the female pupae contamination rate (Mann-Whitney U-test, *U* = 5, *P* = 0.1429) and male pupae production efficiency (Mann-Whitney U-test, *U* = 9, *P* = 0.5317) between the Wol-unit and the IAEA-unit (Table 3). As shown in Table 4, HC males exhibited equal mating competitiveness to GUA males when competing for GUA females in large cages regardless of the rearing unit used (Mann-Whitney U-test, *U* = 3, *P* = 0.7000).

**Table 3:** Pupae production and sex separation efficiency by using the entire larval rearing units

Parameter	Wol-unit	IAEA-unit
Number of trays per rack	40	50
Estimated number of male pupae per tray^a^	2459	7144
Estimated male pupae production per unit (10^5^)^b^	0.98	3.57
Male pupae production per unit (10^5^)	0.89 ± 0.02	3.16 ± 0.11
Female pupal contamination rate (%)	0.5 ± 0.1 a	0.9 ± 0.2 a
Male pupae production efficiency (%)^c^	90.9 ± 2.4 a	88.5 ± 3.0 a

^a^Estimated number of male pupae per tray for Wol-tray and IAEA-tray was obtained from the top, middle and bottom section of larval rearing unit

^b^Estimated male pupae production per unit was calculated as: (Number of trays per unit × Estimated number of male pupae per tray)

^c^Male pupae production efficiency was calculated as: (Male pupae production per unit / Estimated male pupae production)

*Note*: Within each row, values followed by same lowercase letters were not statistically different using Mann-Whitney U-test analysis (*P* > 0.05). All the data in the table were presented as Mean or (Mean ± SE)

**Table 4:** Male mating competitiveness of HC males obtained from different larval rearing units

Male: Male	Fertility (%) (No. of eggs estimated)	Male mating competitiveness index (C)^a^
Fertile control	90.8 ± 0.7% (3074) (Hn)	
Sterile control Wol-unit HC	0 (4550) (Hs)	
Wol-unit HC: GUA	44.5 ± 2.1% (9905) (Ho)	1.05 ± 0.10 a
Sterile control IAEA-unit HC	0 (4164) (Hs)	
IAEA-unit HC: GUA	46.0 ± 2.8% (10,907) (Ho)	0.99 ± 0.12 a

^a^C: Male mating competitiveness index, calculated as: C = [(Hn – Ho)/(Ho – Hs)] × (N/S), where N and S were the numbers of fertile and sterile males

*Note*: Within each column, values followed by same lowercase letters were not statistically different using Mann-Whitney U-test analysis (*P* > 0.05). All the data in the table were presented as Mean or (Mean ± SE)

*Abbreviations*: *Hn* mean egg hatch rate of fertile control cages, *Hs* mean egg hatch rate of sterile control cages, Ho mean egg hatch rate of competitive mating cages

## Discussion

In the present study, we tested two larval rearing units (Wol-unit and IAEA-unit) for their potential application for *Ae. albopictus* larval mass-rearing in support of the establishment of a medium-scale mosquito facility for SIT/IIT strategies. Our study shows that both of these larval rearing systems support high pupae production and good male adult quality under their respective optimized rearing density. Compared to the Wol-unit, the IAEA-unit is recommended for *Ae. albopictus* larvae mass-rearing in a medium-sized mosquito facility, with almost 2.59 times more male pupae production that can be achieved in just 0.7 m^2^ of insectary space.

Previous studies have reported that the larval rearing density would have an impact on the larval development speed of *Ae. albopictus*, with prolonged developmental time resulting from higher rearing densities, possibly due to the resource competition among the larvae [24, 25]. The prolonged developmental time was correlated with fewer pupae produced within a defined time in our study. Similar results were also achieved in the Wol-tray with the higher rearing density of 3.6 larvae/cm^2^ when compared to the other two lower rearing densities (Table 1). However, this pattern is not observed in the IAEA-tray even when the larvae were reared at this high density (Table 1). The difference may be caused by the structure of the tray as the larger size tray may bear a higher larval rearing density, but this needs further study. Male pupae/adult production is an important parameter for a mosquito factory, and this parameter is associated with larval rearing. Thus, selecting an optional larval rearing density resulting in more male pupae within a limited time is recommended. Based on the three tested larval rearing densities in this study, the favorable density is 18,000 L_1_ for IAEA-tray and 6600 L_1_ for Wol-tray (Table 1). *Ae. albopictus* males usually develop faster than females (due to protandry) because females need more energy to develop their organs [24, 25]. Our results also show that 80–90% of the total male pupae production and only 20–30% total female pupae production was achieved at 34 h after the pupation period began for both trays under the three rearing densities tested (Table 1). In the view of large operational programs (which require large mass-rearing mosquito facilities) and in order to reduce labor and costs, it is crucial to determine the optimal time to separate pupae from larvae and not to repeat the operation 2 times (as was done during our experiment). In this study, we clearly demonstrate that 34 h after the pupation process started, almost 85% of the total male pupae production is obtained under the optimized rearing density in both the Wol-tray and the IAEA-tray (Table 1). In addition, even after sex separation, male pupae still need to go through several processes such as irradiation and packaging before the adults emerge. With a separation time at 34 h (obtained in our study), enough time is left to perform the later steps of production.

Water temperature is an important parameter for the development of larvae. Balestrino et al. [25] reported that the optimum water temperature for *Ae. albopictus* is between 26 and 28 °C. The structure of larval rearing units would affect the water temperature when tested in otherwise same conditions [25, 28]. In this study, we found that the average water temperature of the IAEA-trays was approximately 1.4 °C higher than the Wol-trays, when stacked within the IAEA-unit (Table 2). This might be due to the reduced gap between the larval trays when stacked within the IAEA-unit (3 cm) which can reduce evaporative cooling of the water and maintains the humidity of the entire IAEA-unit [25, 28]. Apart from the structure of the larval rearing unit, the height of the larval rearing unit can also affect the water temperature with higher water temperatures observed in the higher layers (Table 2). The warmer air rises and the cooler sinks down, causing the difference of water temperature observed at different levels of the larval rearing units. This gradient difference may be minimized by establishing an air circulation system in the larval rearing room, but this needs further study. Our study on larval rearing density and rearing position in terms of height has limitation, for example, the replication for these two experiments is small, which reduces the robustness of the results statistically.

Sex separation (female elimination) is essential for population suppression by either classical SIT, or genetic, transgenic or symbiont-based technologies [12, 13, 16]. The most reliable method is to develop a genetic sexing strain (GSS) for SIT programs similar to the one available for the Mediterranean fruit fly *Ceratitis capitata* (the Vienna 8 GSS) [32]. However, such a GSS is currently not available for *Ae. albopictus* [16]. Based on the behavioral differences between male and female adults, one of the separation methods is offering blood meals mixed with insecticide or toxicants [33]. For *Ae. albopictus*, a stainless steel sieve can be used to separate male and female pupae based on their size difference [25]. This separation method requires the uniform rearing of larval stages to exploit and maximize the size difference between male and female pupae; otherwise, this might lead to either reduced yield of male pupae or an increased rate of female contamination [28]. It is reported that a reduced female contamination rate can be achieved by making the pupae pass through several sieves of different size, but this method might be not only inefficient but may also cause stress and damage to male pupae [28]. Currently, a modified Fay-Morlan sorter is used for sex separation for *Ae. albopictus* in the Wolbaki facility and the female pupae contamination rate is around 1% for both the Wol-unit and the IAEA-unit (Table 3). It is reported that the maximum acceptable female contamination rate for mosquito SIT-based strategies is less than 1%; however this value should be reduced further when the control strategy is tested in an endemic area of mosquito borne diseases [28, 34]. Previous studies have shown that the combination of SIT and IIT strategies to control *Ae. albopictus* using the HC strain is the safest and a highly effective approach [12, 19, 20], since HC females also inhibit the replication of both dengue and Zika virus (unpublished data) and have reduced ability to transmit diseases in the event of an accidental release of females in an endemic zone. Thus, this female pupae contamination rate when using both Wol-unit and IAEA-unit is acceptable for this integrated approach, contrary to other field trials where non-*Wolbachia* based approaches are employed. Additional quality control measures prior to male adult releases can further reduce the female adult contamination rate, such as draining the water from the release bucket of male adults in advance, thus reducing the emergence of female pupae (female pupae emerge later than male pupae) or providing toxicant-spiked blood meals to females.

The average of male pupae production from the IAEA-unit was 3.16 × 10^5^, approximately 2.54 times higher than that of the Wol-unit (Table 3), while the space requirements for these two units is the same. The comparison between the Wol-unit and the IAEA-unit for the production of one million *Ae. albopictus* HC males per week is also shown in Additional file 1: Table S1. For a mosquito facility, it is the aim to maximize male production (pupae or adults) while minimizing space requirements, thereby improving the mass-rearing efficiency and reducing overall costs. With the sieve sorting method, Balestrino et al. [25] estimated that the IAEA larval rearing unit could produce 1.0 × 10^5^ male pupae on average, however, by using the glass separator, our results show that male pupae production can be 2.16 times higher, which indicates the male production also greatly depends on the pupae sorting methods. Several factors, including the sex separation method, efficiency (separation time and reliability), larval diet and water temperature, could be used to explain this difference. The success of any large scale suppression program depends on the capacity of laboratory mass-reared and sterilized males to compete with wild males to mate with wild females [35]. The current study has indicated that HC males show equal mating competitiveness compared to wild type GUA males at a 1:1 release ratio in the large cages (Table 4), regardless whether they were produced in the Wol-unit or the IAEA-unit. Our results are consistent with our previous study that HC males reared in the laboratory at small scale are competitive compared to GUA males and are able to successfully mate with GUA females [20].

Previous studies have found that the presence of *Wolbachia w*Pip does not have a negative impact on the fitness of the artificially triple-infected HC strains regarding female fecundity and fertility, developmental speed and adult longevity when compared to the wild type *Ae. albopictus* [22]. Both the male production capacity (pupae and adults) and male mating competitiveness have a significant impact on the population suppression technique. The current study clearly indicates that the IAEA larval rearing unit shows high efficiency in terms of the production of males, and resulting high quality insects and is thus applicable for medium-sized mosquito facilities (Additional file 1: Table S1). To improve the male pupae production per larval rearing unit, Wolbaki has generated the second generation of larval rearing unit with two separated columns which can hold 100 trays in total and rear ~1.5 million larvae in less than 1 m^2^ space. Further studies will be performed to assess this new developed larval rearing unit to mass-rear *Ae. albopictus* in larval stages.

## Conclusions

Two larvae rearing units were tested to assess their potential use to mass-rear the larval stages of *Ae. albopictus* in a standardized medium-scale mosquito facility for mosquito control strategies. We compared the effects of larval densities and tray size/position on larval survival, male pupae production, female pupae contamination and male mating competitiveness by using these two larvae rearing units. The results indicate that both the Wol-unit and IAEA-unit are suitable for mass-reared *Ae. albopictus* larvae. The IAEA-larvae mass-rearing unit is considered to be suitable for application in a medium sized mosquito facility due to its high male pupae production and minimal space requirements (Additional file 1: Table S1).

## Additional file

Additional file 1: Table S1. Comparison between the Wol-unit and the IAEA-unit for the production of one million *Aedes albopictus* HC males. (DOCX 16 kb)

## Abbreviations

FAO: Food and Agriculture Organization
GUA: Double *Wolbachia*-infected *Aedes albopictus*
HC: Triple *Wolbachia*-infected *Aedes albopictus*
IAEA: International Atomic Energy Agency
IIT: Incompatible Insect Technique
IPCL: Insect Pest Control Laboratory
SIT: Sterile Insect Technique
WHO: World Health Organization
Wol: Wolbaki
