# Supplementary material for: Establishment of a medium-scale mosquito facility: optimization of the larval mass-rearing unit for Aedes albopictus (Diptera: Culicidae)
Source: Parasit Vectors. 2017 Nov 13;10:569. doi: 10.1186/s13071-017-2511-z (PMC5683581; doi:10.1186/s13071-017-2511-z)
Supplement: Additional file 1: Table S1. — Comparison between the Wol-unit and the IAEA-unit for the production of one million Aedes albopictus HC males. (DOCX 16 kb) [file 13071_2017_2511_MOESM1_ESM.docx]

Table S1 Comparison between the Wol-unit and the IAEA-unit for the production of one million *Aedes albopictus* HC males.

| Parameter | Wol-unit | IAEA-unit |
| --- | --- | --- |
| Dimensions (m, L * W * H) | 0.97 × 0.70 × 1.85 | 0.78 × 1.2 × 2.10 |
| Ground area per unit (m^2^) | 0.68 | 0.94 |
| Quantity (unit) | 11.2 | 3.2 |
| Total space (m^2^) | 7.6 | 3.0 |
| Labor - Adding water | Manual operation | Semi-automatic operation |
| Labor - Pupae/Larvae collection | Manual operation | Semi-automatic operation |
| Labor - Cleaning | Manual operation | Semi-automatic operation |
| Price | Cheep | Expensive |
| Application | Small size facility | Medium size facility |
